# Visualization of terahertz surface waves propagation on metal foils

**DOI:** 10.1038/srep18768

**Published:** 2016-01-05

**Authors:** Xinke Wang, Sen Wang, Wenfeng Sun, Shengfei Feng, Peng Han, Haitao Yan, Jiasheng Ye, Yan Zhang

**Affiliations:** 1 Department of Physics, Capital Normal University, Beijing Key Laboratory of Metamaterials and Devices, and Key Laboratory of Terahertz Optoelectronics, Ministry of Education, Beijing, 100048, P.R. China; 2 Department of Physics, Harbin Institute of Technology, Harbin, 150001, P.R. China

## Abstract

Exploitation of surface plasmonic devices (SPDs) in the terahertz (THz) band is always beneficial for broadening the application potential of THz technologies. To clarify features of SPDs, a practical characterization means is essential for accurately observing the complex field distribution of a THz surface wave (TSW). Here, a THz digital holographic imaging system is employed to coherently exhibit temporal variations and spectral properties of TSWs activated by a rectangular or semicircular slit structure on metal foils. Advantages of the imaging system are comprehensively elucidated, including the exclusive measurement of TSWs and fall-off of the time consumption. Numerical simulations of experimental procedures further verify the imaging measurement accuracy. It can be anticipated that this imaging system will provide a versatile tool for analyzing the performance and principle of SPDs.

With the understanding of the terahertz (THz) radiation located between the microwaves and infrared regions, THz sensing and imaging technologies are being progressively perfected and applied in enormous research and industrial fields, such as carrier transportation in semiconductors^1^,^2^, spectral analysis to a wide range of materials^3^, pharmaceutical quality testing^4^,^5^ and security inspection^6^. The advancement of surface plasmonic devices (SPDs), which can localize electro-magnetic oscillations on a metal foil and achieve kinds of unexpected diffraction functions, immensely prompts the miniaturization of THz systems and enhances the feasibility of THz technologies. Exploring the underlying mechanisms of these special diffraction phenomena, such as resonant filtering^7^, negative refraction^8^, extraordinary transmission^9^, is very pivotal for designing functions of SPDs. Therefore, the requirement of measurement means is very urgent for investigating the coupling and evolution processes of a THz surface wave (TSW).

Similar to the measurement techniques in visible frequencies, including scanning near-field optical microscopy, fluorescence imaging and leakage radiation imaging^10^,^11^,^12^, the information of a TSW also requires to be detected in the near field zone of a sample surface. Currently, the detection methods in the THz frequency range are developed on the basis of the traditional THz time domain spectroscopy, which mainly include scanning near-field THz microscopes (SNTM) utilizing a photoconductive antenna^13^,^14^,^15^ or an electro-optic crystal^16^,^17^,^18^,^19^. A photoconductive antenna can detect the spatial derivative of a TSW and the signal obtained by an electro-optical crystal is directly proportional to the field of a TSW. Advantages of both techniques are the simultaneous acquirement of the amplitude and phase of a TSW due to the coherent measurement mode and the sub-wavelength spatial resolution. However, a two-dimensional raster scan is necessary in both techniques, which results in a big time consumption. S. Wang *et al.* proposed a line scan THz imaging method to measure the Gouy phase shift of a focused TSW for reducing the experimental time, but the imaging system still needs a one-dimensional raster scan^20^.

In this work, a THz digital holographic imaging system is utilized to measure the propagation of TSWs on a metal foil. A <100> ZnTe crystal is chosen as the sensor to selectively detect the two-dimensional pattern of a TSW. The influence of the transmitted transverse THz component is effectively filtered out. In the imaging system, the movement between the sample and the detector is thoroughly avoided to outstandingly reduce the experimental time and strengthen the measurement stability. Two typical surface plasmonic structures are used to check the practicability of the imaging system. Complex field distributions of the excited TSWs in the time and frequency domains are exactly presented. Corresponding simulation results are also obtained to replicate experimental phenomena.

## Results

**Figure 1:**
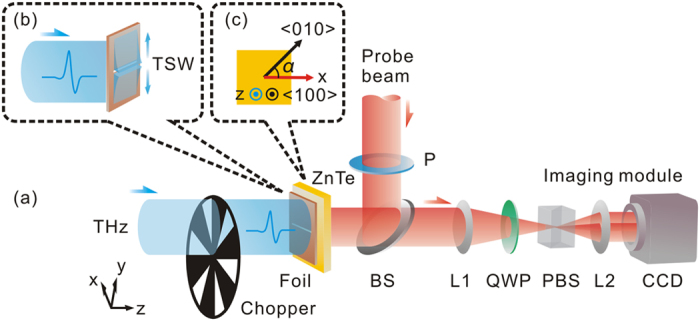
THz digital holographic imaging system. (**a**) Optical configuration of the imaging system. (**b**) Two-dimensional pattern of a TSW excited by the sub-wavelength scale structure of a metal foil. (**c**) Orientation of the ZnTe crystal. The < 100 > direction of the ZnTe crystal is parallel to the z axis. The angle α between the < 010 > direction of the ZnTe crystal and the x axis is 45 degree.

A THz digital holographic imaging system is utilized to characterize the temporal evolution of a TSW. Figure 1(a) shows the schematic of the experimental setup. A y-polarized THz wave impinges onto the surface of a metal foil. Then, the sub-wavelength scale structure of the metal foil provides enough momentum components to achieve the match in wave vector and activate surface plasmon polaritons (SPPs), namely well-known Sommerfeld or Zenneck waves, as shown in Fig. 1(b). Since the THz surface electric field is z-polarized, a <100> ZnTe crystal with a 1 mm thickness is chosen as the sensor to exclusively measure the two-dimensional pattern of the TSW at the exit side of the sample^21^,^22^. The <100> direction of the crystal is parallel to the z axis, as shown in Fig. 1(c). The imaging system allows one to directly coherently measure the THz field in the time domain, so amplitude and phase distributions of TSWs with different frequencies can be obtained simultaneously^23^,^24^.

**Figure 2:**
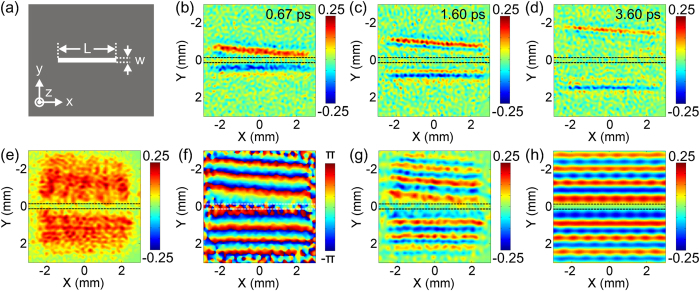
Imaging the linear TSW generated by a rectangular slit structure. (**a**) Schematic of the slit sample. (**b–d**) Time sequences of the TSW distribution on the exit facet of the slit sample at 0.67 ps, 1.60 ps and 3.60 ps. **(e–g**) Amplitude, wrapped phase and real part images of the TSW for the 0.44 THz component. (**h**) Corresponding simulated real part pattern acquired by using the FDTD algorithm.

To check the ability of the imaging system, a simple rectangular slit drilled on a 150 μm thick stainless steel foil is picked, since the structure can excite a linear TSW. Fig. 2(a) gives the schematic of the slit sample. Its length and width are L = 12 mm and w = 250 μm, respectively. The slit sample is mounted close to the surface of the detection crystal as near as possible to effectively measure the TSW with an around 100 μm resolution^25^. The central part of the sample is measured in the experiment and its long side is perpendicular to the THz polarization for efficiently exciting the TSW. The imaging system needn’t one- or two-dimensional raster scan of the sample, so the propagation process of the TSW can be observed only by varying the optical path difference between the THz beam and the probe beam. Figure 2(b–d) show the snapshots of the TSW at three selected times 0.67 ps, 1.60 ps and 3.60 ps. Two dashed lines indicate two edges of the slit. It can be clearly seen that the TSWs appear on both sides of the slit and present an anti-symmetric distribution with respect to the slit symmetry axis. The red and blue colors indicate the amplitude of the TSWs on the two sides of slit, which manifest that the TSWs generated on two sides of silt are equiphase with respect to the slit symmetry axis. The colorbar exhibits that two TSWs have the same amplitudes and inverse polarities. Actually, this phenomenon has been explained in previous references^13^,^26^. The polarity divergence is due to that the normally incident THz electric field polarizes the metal surface on the entrance facet of the sample. The slit obstructs the movement of surface charges to form a potential difference at the opposite slit edges, which gives rise to the different polarized TSWs on two sides of the aperture. As the time delay increasing, the two linear TSWs move away from the slit and their amplitudes gradually attenuate. An 8 ps long TSW trace is recorded with a 0.13 ps time resolution and each frequency component is extracted by performing the Fourier transformation on each pixel. Figure 2(e–g) present the amplitude, wrapped phase, and real part of the complex field for 0.44 THz, respectively. The amplitude image, as shown in Fig. 2(e), depicts that two TSWs have maximum intensities around the slit edges and decay below 1/e of the original amplitude after propagating around 2 mm. The wrapped phase image shown in Fig. 2(f) denotes the propagation directions of the TSWs along positive and negative y axes by monotonically rising phases accompanied with periodic jumps of 2π. It can be seen that behaviors of the TSWs are similar to a damped plane wave in mediums. The real part image of the complex amplitude more explicitly represents the oscillation, propagation, attenuation and different polarities of the TSWs. To further confirm the accuracy of experimental results, a numerical simulation is implemented to replicate the experimental procedure by using the finite-difference-time-domain (FDTD) algorithm. Figure 2(h) shows the simulated real part image of the complex amplitude of the TSW, which is well consistent with the experimental one.

**Figure 3:**
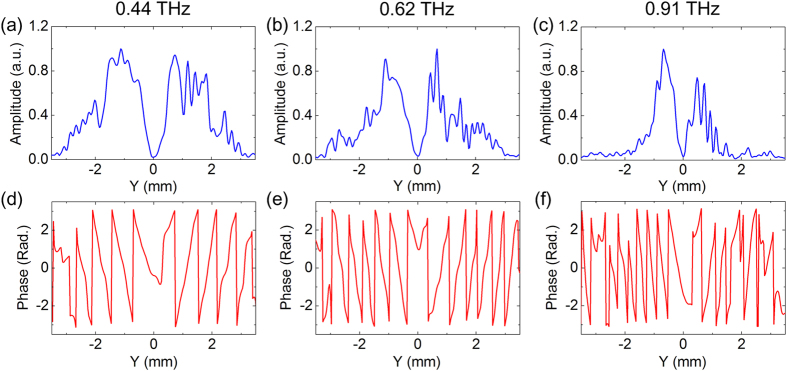
Frequency-dependent characteristics of the linear TSW. (**a**–**c**) Amplitude profiles of the linear TSW along the line x = 0.0 mm for 0.44 THz, 0.62 THz and 0.91 THz. (**d**–**f**) Corresponding wrapped phase plots for different frequencies.

Utilizing the broad bandwidth of the THz source, frequency-dependent characteristics of the TSW can be easily determined. Three slices along the line x = 0 mm from the complex field patterns at 0. 44 THz, 0.62 THz and 0.91 THz are selected out and their normalized amplitudes and wrapped phases are plotted in Fig 3(a–f), respectively. According to the amplitude distributions, propagation distances of three TSWs are evaluated as 2.0 mm, 1.3 mm and 0.5 mm for 0.44 THz, 0.62 THz and 0.91 THz, respectively. Here, the propagation distance is defined as the length between positions of the maximal amplitude and its 1/e. The experimental results manifests that the TSW with a higher frequency has bigger energy loss due to a stronger field confinement to the interface^27^. From wrapped phase plots, the phase increasing tendencies indicate the traveling directions of the TSWs on two sides of the slit. Furthermore, wave numbers of the three TSWs can be calculated as 92.7 ± 6.1 rad/cm, 126.5 ± 11.7 rad/cm and 195.5 ± 24.5 rad/cm using 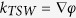, where *φ* is the phase of the TSW. Therefore, the phase velocities of three TSWs can be derived as 2.99 ± 0.21 × 10^8^ m/s, 3.07 ± 0.31 × 10^8^ m/s and 2.93 ± 0.42 × 10^8^ m/s by comparing 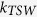 and the vacuum wave number 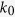. The calculation results show that phase velocities of the three TSWs are very close to the light speed in the vacuum. The phenomenon is in agreement with the expectation, because the dispersion curve of a TSW is very similar to that of a traveling light wave in the free space. Additionally, with increasing frequencies, the measurement errors of the wave numbers and phase velocities gradually intensify. It is attributed to the fact that the sharper phase variation and the weaker intensity of a higher frequency component result in the severer phase error.

**Figure 4:**
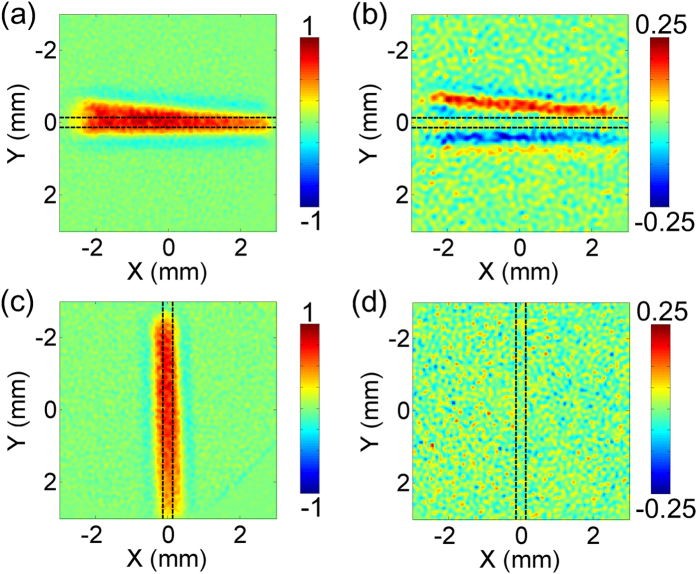
Influence of the orientation of the rectangular slit to the excited TSW. (**a**,**c**) Peak amplitude images of emerging transverse THz components from the rectangular slit aligned perpendicular and parallel to the incident THz polarization. (**b**,**d**) Corresponding amplitude images of the transient TSWs at 0.67 ps time delay.

To corroborate the precision of the measured TSW, the slit sample is rotated 90 degree and is imaged again. For better overview, the transmitted transverse THz field with a linear polarization along the y direction is also measured by replacing the sensor with a <110> ZnTe crystal. The thickness of the crystal is also 1 mm. It should be noted that the <110> and <100> ZnTe crystals with the identical thickness have the same detection efficiencies^28^. Figure 4(a,c) give the temporal peak amplitude images of the transverse THz components transmitted through the slit sample aligned perpendicular and parallel to the THz polarization. The red parts correspond to the emerging THz waves from the aperture, which are a little larger than the size of the aperture due to a slight diffraction influence. In addition, the amplitude of the transverse THz component is about 4 times stronger than the TSW. Figure 4(b,d) show corresponding amplitude patterns of the transient TSW at the time delay of 0.67 ps. It is obvious that there is no TSW signal in Fig. 4(d), which demonstrates that TSWs cannot be excited for TE polarization and the measurement result is not disturbed by the out-going transverse THz component. The advantage of the imaging system is very crucial for exactly characterizing temporal properties of a TSW.

**Figure 5:**
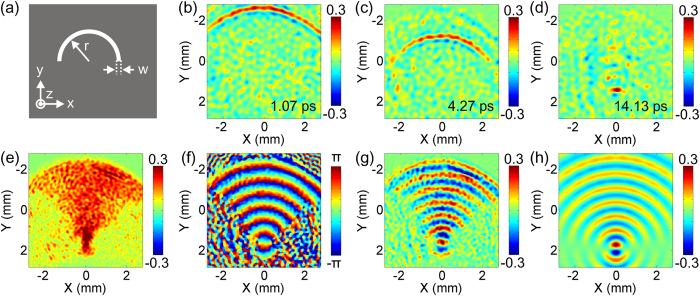
Measurement of the converged TSW excited by a semicircular slit structure. (**a**) Schematic of the slit sample. (**b**–**d**) Transient patterns of the TSW at selected three times of 1.07 ps, 4.27 ps and 14.13 ps. (**e**–**g**), Reconstructed amplitude, wrapped phase and real part distributions of the TSW at 0.44 THz. (**h**) Corresponding numerical simulation of the real part pattern.

As another paradigm, a semicircular slit with a r = 6 mm radius and a w = 270 μm width fabricated on a 150 μm thick stainless steel foil is imaged to observe its focusing function of TSWs^20^,^29^, as shown in Fig. 5(a). The generated TSWs around the edge of the semicircular slit have the same phases after excitation by a y-polarized THz field, so that the TSWs can converge to a spot and the semicircular slit can be viewed as a plasmonic lens. Figure 5(b–d) exhibit the time sequences of the longitudinal THz field at 1.07 ps, 4.27 ps and 14.13 ps, which clearly presents a crescent TSW and its focusing process. By performing the Fourier transformation, the complex field information of the TSW in the frequency domain is acquired. The amplitude, wrapped phase and real part of the complex amplitude for 0.44 THz are extracted and mapped in Fig. 5(e–g). These images intuitively illustrate the focusing function of the semicircular slit. To ensure the accuracy of measurement results, a numerical simulation is also implemented using the FDTD algorithm. The calculated real part of the complex amplitude for 0.44 THz is shown in Fig. 5(h), which reproduces the experimental phenomenon quite well. The feasibility of the imaging system is demonstrated again.

## Discussion

Merits and drawbacks of the THz digital holographic imaging system are discussed for the temporal measurement of a TSW. With the method utilized here, the measurement stability is surely enhanced and the time consumption is significantly reduced due to that the position between the sample and the sensor is stationary in the experiment. According to the imaging region, pixel size, scan time window and temporal resolution, the experimental time of the proposed method is less than 1 hour and that of the SNTM is estimated to be about two days for the same measurement. The proposed imaging technique undoubtedly outperforms previous ones. However, it seems there is still much room left for improving the imaging system. Actually, the signal to noise (SNR) of the system is greatly limited because the lock-in technique cannot be applied^30^. A promising avenue is to increase the intensity of the THz radiation source. Some novel THz generation materials fuel a hope for solving this problem, such as LiNbO_3_, DAST, DSTMS^31^,^32^. Furthermore, the spatial resolution of the proposed method is virtually lower than that of SNTM^13^,^18^,^33^. To overcome this barrier, the thickness of the sensor can be reduced to avoid the diffraction influence of THz waves in the detection crystal. F. Blanchard *et al.* have reported that imaging with a 14 μm resolution was achieved by using a 20 μm thick LiNbO_3_ detection crystal^34^. Unfortunately, an excessive reduction of the crystal thickness will deteriorate the SNR of the imaging system. Therefore, there is still a compelling need to THz detection materials which are sensitive to the THz radiation and suitable for the measurement of a TSW.

To conclude, the ability of a THz digital holographic imaging system is overall demonstrated for measuring TSWs. The propagation process of a TSW in the time domain can be directly coherently obtained with enough spatial and time resolutions. Two-dimensional complex field patterns of TSWs excited by two typical plasmonic structures, including rectangular and semicircular slits, are precisely reconstructed. It is expected that this novel technology can provide a powerful characterization table for further designing and improving SPDs in the THz frequency range.

## Methods

### Measurement method

An impinging laser pulse with a 100 fs pulse duration and an 800 nm central wavelength is split into two beams which are pump and probe beams for exciting and detecting the THz radiation. Firstly, the pump beam with an 890 mW average power is expanded by a concave lens with a 25 mm focal length. Then, the pump beam illuminates a <110> ZnTe crystal with a 3 mm thickness to launch the THz radiation due to the optical rectification. The generated THz wave with a linear polarization along the y direction is collimated by a parabolic mirror with a 75 mm focal length and its diameter reaches approximately 20 mm (is not shown in Fig. 1). The THz beam is incident on the surface of a metal foil to excite the surface electro-magnetic oscillation. The probe beam with a 10 mW average power and a 15 mm diameter emerges through a polarizer (P) to ensure its linear polarization along the x direction. A 50/50 non-polarizing beam splitter is adopted to reflect the probe beam into a detection crystal (a <100> ZnTe crystal with a 1 mm thickness). The <100> direction of the detection crystal is parallel to the z axis. The angle α between the <010> direction of the detection crystal and the probe polarization is aligned as 45 degree for effectively acquiring a TSW^22^, as shown in Fig. 1(c). The probe polarization is only modulated by the longitudinal THz field along the z direction to independently carry the information of the TSW, because the detection crystal is blind to the x and y components of the THz field. The probe beam, reflected from the left surface of the detection crystal, is sent to an imaging module which composes of a quarter wave plate (QWP), a Wollaston prism (PBS), two lenses (L1 and L2) and a CCD camera with a 4 Hz frame rate for measuring the ellipticity of the probe beam. A machined chopper is inserted onto the THz beam and is synchronized with the CCD camera. The imaging module operates the balanced electro-optic detection and the dynamic subtraction technique to extract the two-dimensional THz information^23^,^24^. The imaging area is 5 mm × 5 mm and the pixel size is 32 μm. To observe the propagation of a TSW, the time delay between the THz beam and the probe beam is successively adjusted. At each scan position, 100 frames are averaged to enhance the SNR of the imaging system.

## Additional Information

**How to cite this article**: Wang, X. *et al.* Visualization of terahertz surface waves propagation on metal foils. *Sci. Rep.* **6**, 18768; doi: 10.1038/srep18768 (2016).
